# First larval description of *Nipponomyia* Alexander (Diptera, Pediciidae) with a new species from South Korea

**DOI:** 10.3897/zookeys.1293.201884

**Published:** 2026-09-25

**Authors:** Sigitas Podenas, Virginija Podeniene, Jekaterina Havelka

**Affiliations:** 1 Institute of Biosciences, Life Sciences Centre of Vilnius University, Sauletekio Str. 7, LT-10257 Vilnius, Lithuania Institute of Biosciences, Life Sciences Centre of Vilnius University Vilnius Lithuania; 2 Nature Research Centre, Akademijos Str. 2, LT-08412 Vilnius, Lithuania Nature Research Centre Vilnius Lithuania

**Keywords:** COI DNA barcoding, crane flies, immature stages, Pediciinae, phylogenetic relationships, species delimitation, taxonomy, Tipuloidea

## Abstract

This study presents the first description of the larval stage of the crane fly genus *Nipponomyia* Alexander, 1924 (Diptera: Pediciidae), based on material from South Korea. In addition, a new species, *Nipponomyia kalbongsanica* Podenas & Podeniene, **sp. nov**., is described, representing the first record of the genus from the Korean Peninsula. Detailed morphological descriptions of adults and larvae are provided, including diagnostic characters of the head capsule, mouthparts, spiracular field, and abdominal structures. The larva is shown to possess a combination of features typical of Pediciinae but also exhibits characters that distinguish *Nipponomyia* from related genera such as *Heterangaeus* and *Tricyphona*. Association of larval and adult stages was confirmed using mitochondrial COI DNA barcoding. Phylogenetic analysis supports this association but reveals incongruence between morphological classification and molecular relationships within the genus, as the new species groups morphologically with the *N. trispinosa* species group but clusters genetically closer to the *N. kuwanai* group. Ecological observations indicate that larvae develop in mud accumulated on stones beneath leaf litter in fast-flowing mountain streams. The study expands knowledge of the diversity, morphology, and biology of Pediciidae and highlights the importance of integrating morphological and molecular data in crane fly taxonomy.

## Introduction

*Nipponomyia* Alexander, 1924 is a small genus of the family Pediciidae comprising 17 described species (Oosterbroek 2026) in the East Palaearctic and the Oriental regions. Within the East Palaearctic region, Japan hosts the highest diversity, with four species. One species has been described from China, and two species are known from the Russian Far East. Until now, no records of *Nipponomyia* have been reported from the Korean Peninsula.

Diagnostic morphological characters of the adult for the genus *Nipponomyia* were originally described by Charles Paul Alexander (1924) and later supplemented in subsequent works (Alexander 1935, 1958). A revision of the Japanese species, including descriptions of two new species, *N. okinawensis* Kolcsár & Kato and *N. yakushimensis* Kolcsár & Kato, was recently provided by Kolcsár et al. (2020). The genus is morphologically characterised by a combination of features, including apparently bare compound eyes (“hairy eyes” are a diagnostic feature for Pediciidae in general), a wing with a conspicuous yellow longitudinal stripe along the anterior margin, and a gonostylus bearing 2–14 black, chitinised spines (Alexander 1924, 1935, 1958). Kolcsár et al. (2020) classified Japanese species into two morphological species groups: the *N. kuwanai* species group, bearing black transverse lines (dashes) in the costal cell, and the *N. trispinosa* species group, characterised by the absence of these lines.

Ten genera of the family Pediciidae are known, predominantly from the Northern Hemisphere. Larvae have been described for only six genera: *Dicranota* Zetterstedt, 1838; *Heterangaeus* Alexander, 1925; *Nasiternella* Wahlgren, 1904; *Pedicia* Latreille, 1809; *Tricyphona* Zetterstedt, 1837; and *Ula* Haliday, 1833 (Alexander 1920; Crisp and Lloyd 1954; Lindner 1959; Brindle 1963, 1967; Savchenko 1983; Krivosheina 2009; Krivosheina and Krivosheina 2011; Oboňa and Starý 2013; Podenas et al. 2014). Larvae of Pediciidae, as among other members of the superfamily Tipuloidea, are hemicephalic and metapneustic. They possess a slightly reduced head capsule and bear two spiracular lobes in the subfamily Pediciinae or five in the subfamily Ulinae. With the exception of the mycetophagous genus *Ula*, pediciid larvae are predatory. Most larvae of Pediciinae develop in aquatic habitats or in wet soil near water, whereas larvae of *Nasiternella* are associated with decaying wood.

The morphology of the larval head capsule is one of the most informative characters for distinguishing members of this family. Typical diagnostic features include the strongly sclerotised head capsule and a toothed hypostoma divided into two parts, connected only at the base of the median teeth (Lindner 1959; Hennig 1973; Savchenko 1986; Krivosheina 2009). Although the presence of two spiracular lobes characterises larvae of Pediciinae, this character also occurs in several genera of the family Limoniidae.

No preimaginal stages of *Nipponomyia* were previously known (Gelhaus and Podeniene 2019). Recent fieldwork in South Korea yielded a larval specimen associated with *Nipponomyia*, enabling the first description of the genus’s immature stages. In this paper, we provide descriptions, illustrations, and ecological notes for the species, and outline morphological features that appear diagnostic for the genus.

In addition to morphological examination, mitochondrial cytochrome c oxidase subunit I (COI) DNA barcoding has become a widely used tool for species identification and delimitation in insects (Hebert et al. 2003; Janzen et al. 2005). The COI barcode region provides a standardised genetic marker that is particularly useful for associating different life stages of the same species when rearing is not possible and when diagnostic morphological characters of immature stages are unknown or insufficient. In Tipuloidea, DNA barcoding has proven especially valuable for linking larvae, pupae, and adults (Podeniene et al. 2023).

Currently, five COI barcode sequences of the genus *Nipponomyia* are available in the GenBank database. Four sequences correspond to Japanese species: *N. kuwanai* (MT874511), *N. trispinosa* (MT874512, MT874513), and *N. pentacantha* (MT874514), whereas one sequence is identified only to the genus level (MW114536). To date, no phylogenetic analysis specifically addressing relationships within *Nipponomyia* has been performed.

## Material and methods

Despite the examination of many museum collections, *Nipponomyia* crane flies from the Korean Peninsula were found only in the collections of the Snow Entomological Museum, University of Kansas, Lawrence, KS, USA (**SMEK**). Other *Nipponomyia* specimens used for comparison were studied in the collections of the Natural History Museum, London, UK (**NHMUK**); Naturalis Biodiversity Center, Leiden, the Netherlands (**NBC**); and the Smithsonian National Museum of Natural History, USA (**USNM**).

In 2012, together with entomologists from the National Institute of Biological Resources, Incheon, South Korea (**NIBR**), we initiated intensive studies of Korean crane flies. Crane flies were collected annually, with fieldwork conducted from early spring to late autumn at various localities throughout the country; tens of thousands of specimens were collected and deposited permanently in the NIBR collections. Most of the adults were collected by the senior author in South Korea (2012–2025) and immature stages by the second author including the larva that is the focus of this paper. All NIBR specimens used in this publication are currently stored temporarily at the Nature Research Centre, Vilnius, Lithuania (**NRC**).

Adult crane flies were collected in various ways, including insect nets, Malaise traps, and light traps (Green-LED and New Jersey (NJ)), but only entomological nets were effective for collecting *Nipponomyia* and not a single specimen was collected by other methods. We obtained 46 adult *Nipponomyia* specimens from Korea for our study. Collected specimens preserved dry were mounted laterally on paper points. Wet specimens were preserved in 96% ethanol (EtOH). Some male wings were slide-mounted in Euparal and photographed. Dissected terminalia were cleared in 10% KOH and preserved in microvials with glycerol. Information on the examined material is given according to journal requirements; therefore, altitudes are presented in the metric system, regardless of the system on the label. For specimens collected by SP and his colleagues, the date on the label is followed by a number in brackets. Every collection event (specific locality and date) was assigned a field number by SP and field notes, database, photographs, and other information was catalogued under that field number. Specimens are arranged by collection date.

The larva was preserved in 70% ethanol. Larval and adult specimens of *Nipponomyia* were associated using COI barcode sequences. This molecular evidence enabled reliable identification of the previously unknown larval stage and confirmed its conspecificity with the adult specimens.

The larval head capsule of *Nipponomyia* was cleaned for almost an hour in hot (~10%) KOH solution, and a temporary slide was made in glycerol-gelatine. Later, details of the head capsule were traced and redrawn by hand. The spiracular field of the larva was excised, and a temporary slide was prepared in glycerol-gelatine.

Crane flies were examined with an Olympus SZX10 dissecting microscope and a Nikon Eclipse Ti microscope. Photographs were taken with a Canon R5 camera through a Canon MP-E 65 mm macro lens and through Mitutoyo M Plan apo 10× and 20× lenses mounted on the same camera. Photographs and measurements of the details of the larval head capsule were taken with a Leica DCM6 microscope and a Leica K3C camera using LAS X Core software. The length and width of the head capsule were measured before placing the larva in KOH solution. The width was measured at the widest point of the head capsule, and the length was measured from the anterior end of the labrum to the end of the head capsule. The length of the larva was measured from the anterior end of the first thoracic segment (head retracted) to the apex of the spiracular lobes.

A phylogenetic tree was constructed based on the mitochondrial COI DNA barcoding region (658 bp) sequence data from one adult male (PZ235368), one larva (PZ235369), and sequences from five specimens representing *Nipponomyia* sp. (MW114536), *N. kuwanai* (MT874511), *N. pentacantha* (MT874514) and *N. trispinosa* (MT874512, MT874513) from GenBank database. Total genomic DNA was extracted from the adult leg and larval abdominal segments using a QIAamp DNA Micro Kit (Qiagen, Hilden, Germany), and samples were incubated with proteinase K overnight at 56 °C. For the ampliﬁcation of partial COI sequences, the primers LCO-1490 and HCO-2198, designed by Folmer et al. (1994), were used. PCR ampliﬁcation was carried out in a thermal cycler (Eppendorf) in 25 µL of reaction mixture, containing 2 µL genomic DNA, 2.5 µL of 10× Taq polymerase buffer with KCl, 2.5 µL of dNTP mix (2 mM), 2.5 µL of MgCl_2_ (2.5 mM), 2.5 µL of each primer (1 µM), 0.2 µL of Taq polymerase (recombinant, 5 u/µL) (Thermo Scientiﬁc), and nuclease-free water to 25 µL (Thermo Scientiﬁc). The cycling parameters were as follows: initial denaturation at 95 °C for 5 min (1 cycle), denaturation at 95 °C for 30 s, annealing at 45 °C for 30 s, extension at 72 °C for 30 s (40 cycles in total), and ﬁnal extension at 72 °C for 5 min (1 cycle). PCR products were visualised on 1.5% agarose gel stained with ethidium bromide, puriﬁed using a GeneJET PCR puriﬁcation kit (Thermo Scientiﬁc), and then sequenced at Macrogen Europe (Amsterdam, the Netherlands). The ampliﬁcation primers were also used as sequencing primers. Before further analysis, DNA sequences for each specimen were aligned in the BioEdit Sequence Alignment Editor (Hall 1999). COI sequences were submitted to GenBank. A maximum likelihood (ML) phylogenetic tree was computed with MEGA 12 software (Kumar et al. 2024) using all sites and an extensive subtree-pruning-regrafting (SPR) heuristic search, with a strong branch swap filter and bootstrap test (1000 replicates). The best-fit model of evolution (GTR+I+G) was selected via model selection option in MEGA 12 (Kumar et al. 2024). Genetic distances between examined species were calculated as Kimura 2-parameter distances (K2P), as implemented in MEGA 12 (Kumar et al. 2024). We used mitochondrial COI sequences of *Ulomorpha longipenis* Kato & Kolcsár, 2020 (OQ404934) and *Dicranota gracilipes* Wahlgren, 1905 (MW459443) as an outgroup. Adult and larval associations were accepted when bootstrap values rose to 95–100% or clustered together in a monophyletic unit. The terminology of adult morphological features generally follows that of Cumming and Wood (2017), while wing venation terminology follows de Jong (2017). We use the terminology of Oosterbroek and Theowald (1991) and McAlpine (1981) for morphological features of the larva.

## Taxonomy

### Nipponomyia

Taxon classificationAnimaliaDipteraPediciidae

Alexander, 1924

09BB9190-BE9B-545A-91A8-AB92ECEEF61D

Nipponomyia Alexander 1924: 158, 159; Alexander 1958: 292; Ishida 1958: 37 (key), 39. Savchenko and Krivolutskaya 1976: 24 (key), 25; Savchenko 1983: 33; Savchenko 1989: 33; Kolcsár et al. 2020: 74–79.

#### Type species.

*Tricyphona kuwanai* Alexander, 1913. Type locality. Japan (East Palaearctic).

#### Examined material.

**Figures 1–14.**
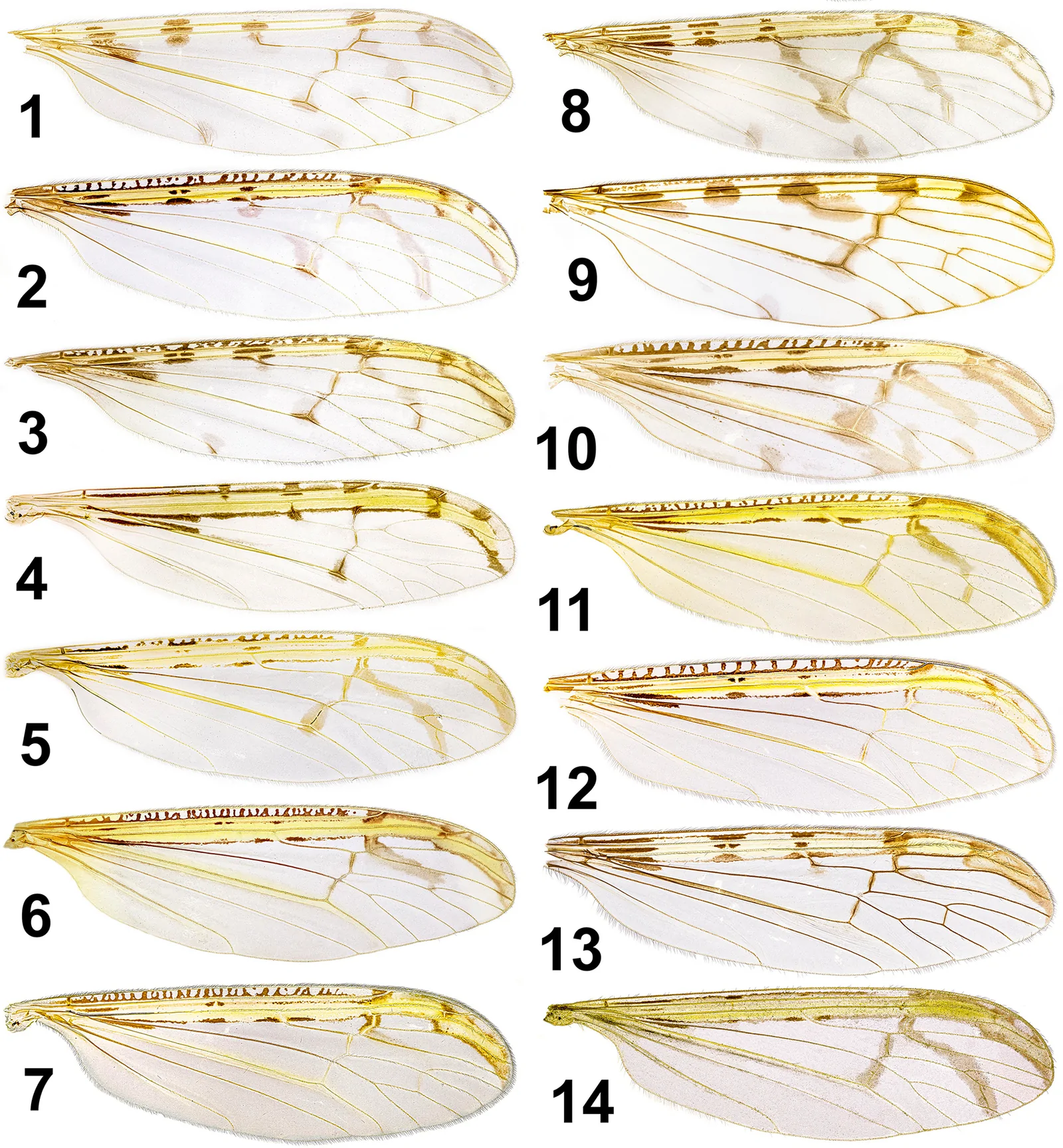
*Nipponomyia* wings. **1**. *N. flavicollis* Edwards, 1933, paratype, female; **2**. *N. joshii* Alexander, 1957, male; **3**. *N. kamengensis* Alexander, 1967, holotype, male; **4**. *N. khasiana* Alexander, 1936, holotype, male; **5**. *N. kulingensis* holotype, female; **6**. *N. kuwanai* (Alexander, 1913), holotype, female; **7**. *N. kuwanai*, male; **8**. *N. mannheimsiana* Alexander, 1969, holotype, male; **9**. *N. nigrocorporis* Alexander, 1944, holotype, male; **10**. *N. novempunctata* (Senior-White, 1922), male; **11**. *N. pentacantha* Alexander, 1958, paratype, male; **12**. *N. symphyletes* (Alexander, 1923), male; **13**. *N. szechwanensis* Alexander, 1935, holotype, male; **14**. *N. trispinosa* (Alexander, 1920), holotype, male.

*N. flavicollis* Edwards, 1933. **Malaysia** • ***Holotype***: ♂ (tip of abdomen broken) (pinned); B. N. Borneo, Mt. Kinabalu, Pakka; [6.08661°N, 116.55858°E]; alt. 3048 m; 26 March 1929; H. M. Pendlebury leg.; NHMUK010397780. ***Paratypes***: • 1 ♀ (slide mounted wing (Fig. 1)); B. No. Borneo, Mt. Kinabalu, Pakka; [37.74946°N, 127.16385°E]; alt. 3048 m; 23 March 1929; at light; USNM • 1 ♀ (pinned); B. N. Borneo, Mt. Kinabalu; [37.74946°N, 127.16385°E]; alt. 2134 m; 27 March 1929; NHMUK • 1 ♀ (pinned); B. N. Borneo, Mt. Kinabalu; [37.74946°N, 127.16385°E]; 28 March 1929; NHMUK • 1 ♀ (pinned); B. N. Borneo, Mt. Kinabalu; [37.74946°N, 127.16385°E]; 29 March 1929; NHMUK • 1 ♀ (pinned); B. N. Borneo, Mt. Kinabalu, Pakka; [37.74946°N, 127.16385°E]; 29 March 1929; NHMUK.

*N. joshii* Alexander, 1957. **Nepal** • ***Holotype***: ♀ (pinned, wing and hind leg slide mounted); Nepal, Simbhanjang Pass; [27.59082°N, 85.08460°E]; alt. 2496 m; 1 October 1956; E. I. Coher leg.; USNM. **India. *Non types***: • 1 ♀ (pinned); Sikkim; F. Schmid leg.; USNM • 1 ex. (pinned); Sikkim; 1959; F. Schmid leg.; USNM • 1 ex. (pinned); Sikkim; 30 April 1959; F. Schmid leg.; USNM • 1 ex. (pinned); Sikkim, Lathong; alt. 2000 m; May 1959; F. Schmid leg.; USNM • 1 ♂, 1 ♀ (on same pin); Sikkim, Lathong; F. Schmid leg.; USNM • 1 ex. (pinned); Sikkim, Lachung; 3 July 1959; F. Schmid leg.; USNM • 1 ex. (pinned); Sikkim, Lachung; 1959; F. Schmid leg.; USNM • 1 ♂, 1 ♀ (on same pin); Sikkim; 1959; F. Schmid leg.; USNM • 1 ex. (pinned); Assam, Khasi-Jaint; F. Schmid leg.; USNM • 1 ♂ (head, wing (Fig. 2), fore and hind legs, and terminalia slide mounted); Sikkim, Mangalbarey; alt. 853 m; 30 April 1959; F. Schmid leg.; USNM • 1 ♂ (antenna, wing, fore, middle and hind legs, and terminalia slide mounted); Assam, Khasi-Jainvia, Serrarim; alt. 1768 m; 3 October 1960; F. Schmid leg.; USNM.

*N. kamengensis* Alexander, 1967. **India** • ***Holotype***: ♂ (pinned, head, wing (Fig. 3), leg and terminalia slide mounted); Assam, North East Frontier Agency, Kameng, Foothills; alt. 2134 m [slide label says 213 m, original description 2134 m]; 5 February 1961; F. Schmid leg.; USNM. ***Paratype***: • 1 ♀ (on same pin as holotype); Assam, North East Frontier Agency, Kameng, Foothills; alt. 2134 m [slide label says 213 m, original description 2134 m]; 5 February 1961; F. Schmid leg.; USNM.

*N. khasiana* Alexander, 1936. **India** • ***Holotype***: ♂ (pinned, wing (Fig. 4), leg and terminalia slide mounted); Assam, Cherrapunji, Khasi Hills; alt. 1219 m; August 1935; S. Sircar leg.; USNM. ***Paratypes***: • 1 ♀ (pinned); Assam, Cherrapunji; alt. 1219 m; August 1935; S. Sircar leg.; NHMUK010397781 • 1 ♀ (pinned); Assam, Cherrapunji; alt. 1219–1524 m; S. Sircar leg.; NHMUK010397781. ***Allotype***: • 1 ♀ (pinned); Assam, Cherrapunji; alt. 1219 m; August 1935; S. Sircar leg.; USNM. ***Non types***: • 1 ♀ (pinned); Assam, Khasi-Jainvia; F. Schmid leg.; USNM • 1 ♀, 1 ex. (on same pin); Assam, Khasi-Jainvia; F. Schmid leg.; USNM • 2 ♀ (on same pin); Assam, Khasi-Jainvia; F. Schmid leg.; USNM • 1 ♀ (pinned); Assam, Khasi-Jainvia; F. Schmid leg.; USNM • 1 ♀ (pinned); Assam, Khasi-Jainvia; F. Schmid leg.; USNM.

*N. kulingensis* Alexander, 1937. **China** • ***Holotype***: ♀ (both antennae, wing (Fig. 5) and hind leg slide mounted); Kuling, Kiangsi; alt. 1000 m; 18 August 1935; O. Piel leg.; USNM. ***Non type***: • 1 ♂ (pinned); Mountain Kuling, Kiu Kiang; 23 July; Y. W. Djou leg.; USNM.

*N. kuwanai* (Alexander, 1913). **Japan** • ***Holotype***: ♀ (wing slide mounted (Fig. 6)); Japan, Tokyo; 7 May 1912; USNM. ***Allotype***: • 1 ♂ (pinned); R. Takahashi leg.; USNM. ***Non types***: • 1 ♂ (pinned, antenna, wing, hind leg, and terminalia slide mounted); Tokyo; 7 April 1919; R. Takahashi leg.; USNM • 1 ♂ (pinned, wing and terminalia slide mounted); Meguro, Tokyo; 15 April 1919; R. Takahashi leg.; USNM • 1 ♂ (pinned, antenna, wing (Fig. 7), two legs, and terminalia slide mounted); Honshiu, Iwate-gun, Iwate-ken; alt. 914 m; 28 June 1935; H. Yamamoto leg.; USNM.

*N. mannheimsiana* Alexander, 1969. **India** • ***Holotype***: ♂ (pinned, antenna, palpus, wing (Fig. 8), both hind legs and distal part of abdomen slide mounted); Assam, NEFA – Kameng. Bomdi La; alt. 2682 m; 15 July 1961; F. Schmid leg.; USNM. ***Allotype***: • 1 ♀ (on same pin as holotype, wing and fore leg slide mounted); Assam, NEFA – Kameng. Bomdi La; alt. 2682 m; 15 July 1961; F. Schmid leg.; USNM.

*N. nigrocorporis* Alexander, 1944. **Indonesia** • ***Holotype***: ♂ (pinned, wing (Fig. 9), fore leg and gonocoxite with gonostyli slide mounted); N. Sumatra, Atjen, Mt. Leuser; alt. 2000–3500 m; 30 January 1937; A. Hoogerwert leg.; USNM.

*N. novempunctata* (Senior-White, 1922). **India** • ***Holotype***: ♂ (pinned); Assam, Shillong; 9 October 1920; R. Senior White leg.; in scrub in shade; NHMUK010397782. ***Non types***: • 3 ♀ (pinned); Assam, Shillong; September – October 1918; I. Fletcher leg.; USNM • 1 ♂ (pinned); Assam; alt. 1524 m; USNM • 1 ♂ (wing (Fig. 10) and terminalia slide mounted); Shillong; 17 August 1914; I. Fletcher leg.; USNM.

*N. pentacantha* Alexander, 1958. **Japan** • ***Holotype***: ♂ (pinned, antenna, fore leg, wing and terminalia slide mounted); Honshu, Mt. Amakazari-Echigo; 25 June 1955; K. Baba leg.; USNM. ***Paratype***: • 1 ♂ (pinned, head, wing (Fig. 11), fore leg, and terminalia slide mounted); Honshu, Mt. Amakazari-Echigo; 26 June 1955; K. Baba leg.; USNM.

*N. symphyletes* (Alexander, 1923). **Taiwan** (as Formosa) • ***Holotype***: ♂ (pinned, wing and terminalia slide mounted); Musha; alt. 1067 m; 20 August 1921; T. Esaki leg.; USNM. ***Non types***: • 2 ♂ (pinned, antenna, wing (Fig. 12), leg, and terminalia of 1 ♂ slide mounted); Hassensan; alt. 1707 m; 22 October 1929; S. Issiki leg.; USNM • 1 ♂ (pinned); Hassensan; alt. 800 m; 26 June 1934; Gressit leg.; USNM.

*N. szechwanensis* Alexander, 1935. **China** • ***Holotype***: ♂ (pinned, wing (Fig. 13), hind leg and gonocoxite with gonostylus slide mounted); Szechwan, Kwanhsien; alt. 610–1219 m; 15–31 May 1933; D. C. Grahan leg.; USNM.

*N. trispinosa* (Alexander, 1920). **Japan** • ***Holotype***: ♂ (two wings (Fig. 14), legs and terminalia mounted on three slides); A. Nohira leg.; USNM. ***Non types***: • 2 ♂ (pinned, two wings and terminalia of 1 ♂ slide mounted); Honshiu, Otsu; 9 October 1915; N. Annandale leg.; dancing in a small swarm about 1.5 ft above the ground in a shady lane at dusk; USNM • 1 ♂ (pinned, antenna, wing, fore leg and terminalia slide mounted); Honshu, Kure-Aki; 16 October 1955; K. Baba leg.; USNM • 1 ♂ (head, thorax and abdomen slide mounted); Honshu, Gifu; 15 November 1931; S. Kariya leg.; USNM • 2 ♂ (three wings and four legs slide mounted); Honshu, Gifu; 15 November 1931; S. Kariya leg.; USNM • 1 ♂ (pinned, head, wing, hind leg and terminalia slide mounted); Shikoku, Mt. Sara, Iyo; alt. 800 m; 6 September 1953; T. Yano leg.; USNM • 1 ex. (pinned); Honshu, Gifu; alt. 800 m; 15 September 1921; Takeuchi leg.; USNM. **Russia** • 1 ♂ (pinned); Kunashir, Tretjakovo; 6 September 1972; E. Savchenko leg.; NBC.

#### Diagnosis.

Eye glabrous, with only a few scattered submarginal setae. Antenna short, not exceeding the length of the head. Wing distinctly patterned, with a yellow stripe extending along the anterior margin, usually bordered by narrow dark brown margins or spots; a dark area usually surrounds the base of *Rs* and extends to the distal part of *M*, reaching the cord, which is also surrounded by a dark area. Another oblique dark stripe extends through the base of cell *r_3_* and the basal part of *R_5_*, and continues through the base of cell *m_1_*, sometimes reaching the wing margin at the tip of *M_3_*. In this case, cross-vein *m-m* is very oblique, reaching *M_3_* close to the wing margin. Male terminalia with elongate gonocoxite bearing a large apical lobe armed with numerous small black spines. This lobe is sometimes referred to as the second gonostylus, or the outer lobe of a single gonostylus. One pair of gonostyli present. Gonostylus with a large rostral lobe armed with a few strong, black, often arched spines. Ovipositor elongate; hypogynial valve with a few strong setae curved caudad along the dorsal margin.

#### Description.

Medium-sized Pediciidae crane flies with body length 10.0–16.0 mm and wing length 8.5–13.5 mm. General body colour is yellow (Fig. 16), often with black markings; only *N. nigrocorporis* from Sumatra is a black species.

***Head***. Slightly extended posteriorly, covered with erect setae; vertex wide in both sexes. Eyes generally without setae between ommatidia, except for a very few at the margin. Antenna (Fig. 17) short, reaching slightly beyond the frontal margin of the presutural scutum when bent backwards. Scape up to 2× as long as the pedicel, usually less than that. Flagellum 11–14-segmented, flagellomeres slightly elongate or oval, covered with short pubescence, longest verticils just slightly exceeding the length of the respective segments. Apical antennomere comparatively large, longer than the preceding segment. Rostrum short.

***Thorax***. Dorsum of the thorax with distinct polished black spots or uniformly yellow, except completely black in *N. nigrocorporis*. Cervical sclerite spindle shaped. Pronotum large, bearing erect setae dorsally. Mesonotal prescutum with an indistinct pseudosutural fovea, often with a few distinct dark spots, or uniformly brownish or yellow, sometimes with indistinct longitudinal stripes. Katepisternum without setae. Meron very small, thus middle and posterior coxae are close to each other. Wing (Figs 1–14, 18) moderately wide to narrow, 3.2–4.0× as long as wide, similar in both sexes. Wing with a distinct pattern of dark spots, dots, and yellow stripes. Costal cell often with numerous small dots, sometimes fewer but elongate spots or a complete stripe. Veins *Sc* and *R* surrounded by a distinct yellow area reaching the wing margin, the yellow area along *Cu* is less distinct, but usually reaches the wing margin along *CuA.* A dark area surrounds the cord, a dark oblique line starts at the base of *R_2+3_* and extends, depending on species, either a short distance along the *R* branches, reaching *m-m*, or even to the wing margin. Venation: bases of *R*, *M* and *Cu* veins reach each other at the base, thus the *arculus* is very short or missing. Vein *Sc* long, reaching the wing margin at or often beyond the branching point of *R_2+3_* and *R_4_*, *sc-r* is located a long distance before the origin of *Rs*, in some species approximately at the middle between the origin of *Rs* and the humeral vein, in other closer to the origin of *Rs*; *R_1_* up to 4× as long as *R_2_*. Radial sector long, several times as long as *m-cu*, branches into *R_2+3+4+5_* and *r-m*. Cell *r_4_* with a short stem. Discal cell very long, up to 5× as long as wide, occasionally absent due to reduction of vein *m-m* (*N. khasiana* Alexander, 1936). Cell *m_1_* without a stem, cross vein *m-m* often on *M_2_*. *M_4_* usually branches out before the middle of the discal cell. Cross-vein *m-cu* usually at, sometimes a short distance from, the branching point of *M.* Veins *CuP* and *A_1_* strongly divergent, *CuP* nearly straight, *A_1_* slightly arched at the apex and reaching the wing margin before or approximately at the level of the base of *Rs.* Anal cell long and narrow. Wing squama with a few setae. Tibial spurs short, but present on all legs. Claw simple, without spines or teeth.

**Figures 15–18.**
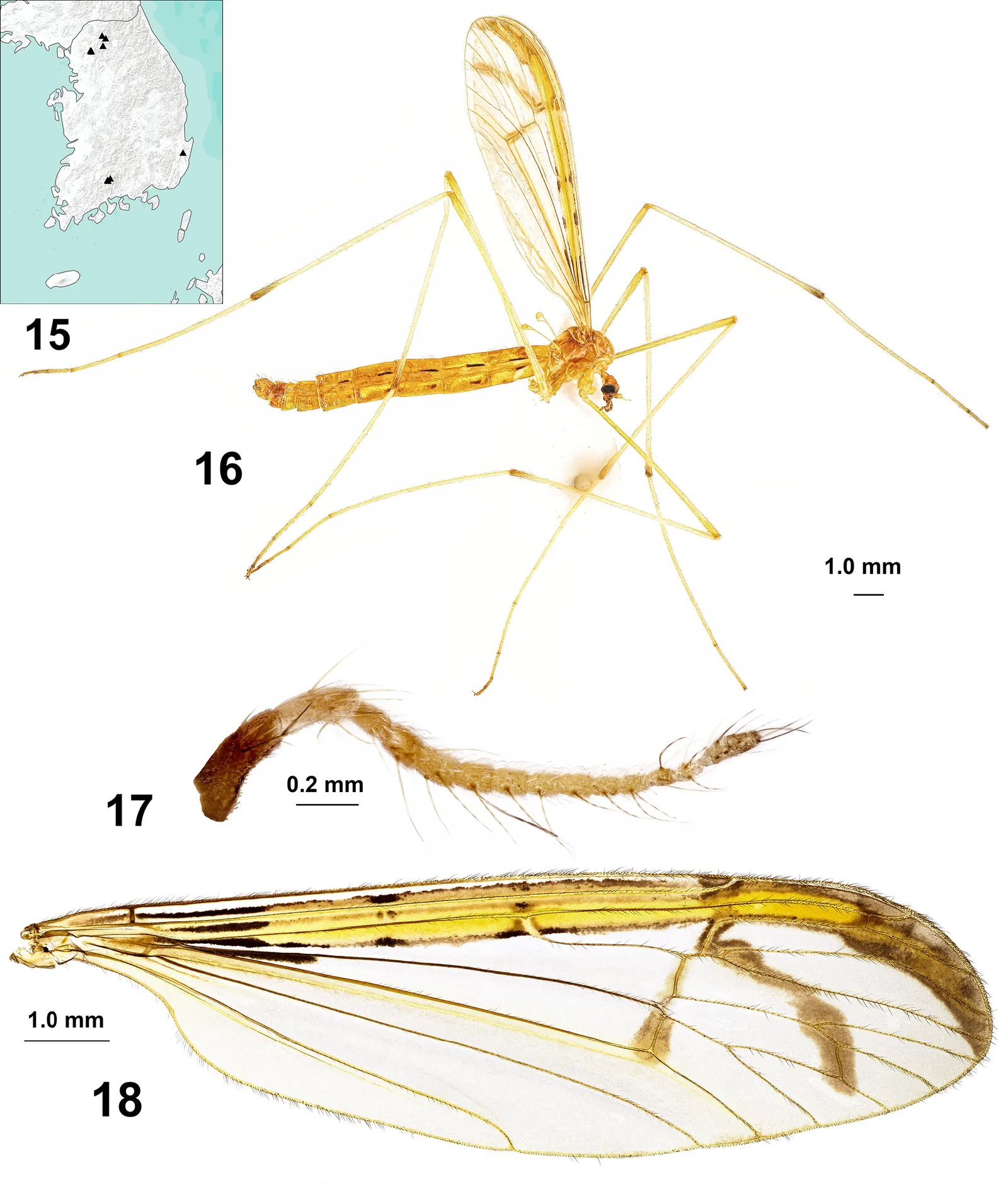
*Nipponomyia kalbongsanica* Podenas & Podeniene, sp. nov. **15**. Sampling localities; **16**. Male, holotype, general view; **17**. Antenna, paratype, male; **18**. Wing, paratype, male.

**Figures 19–24.**
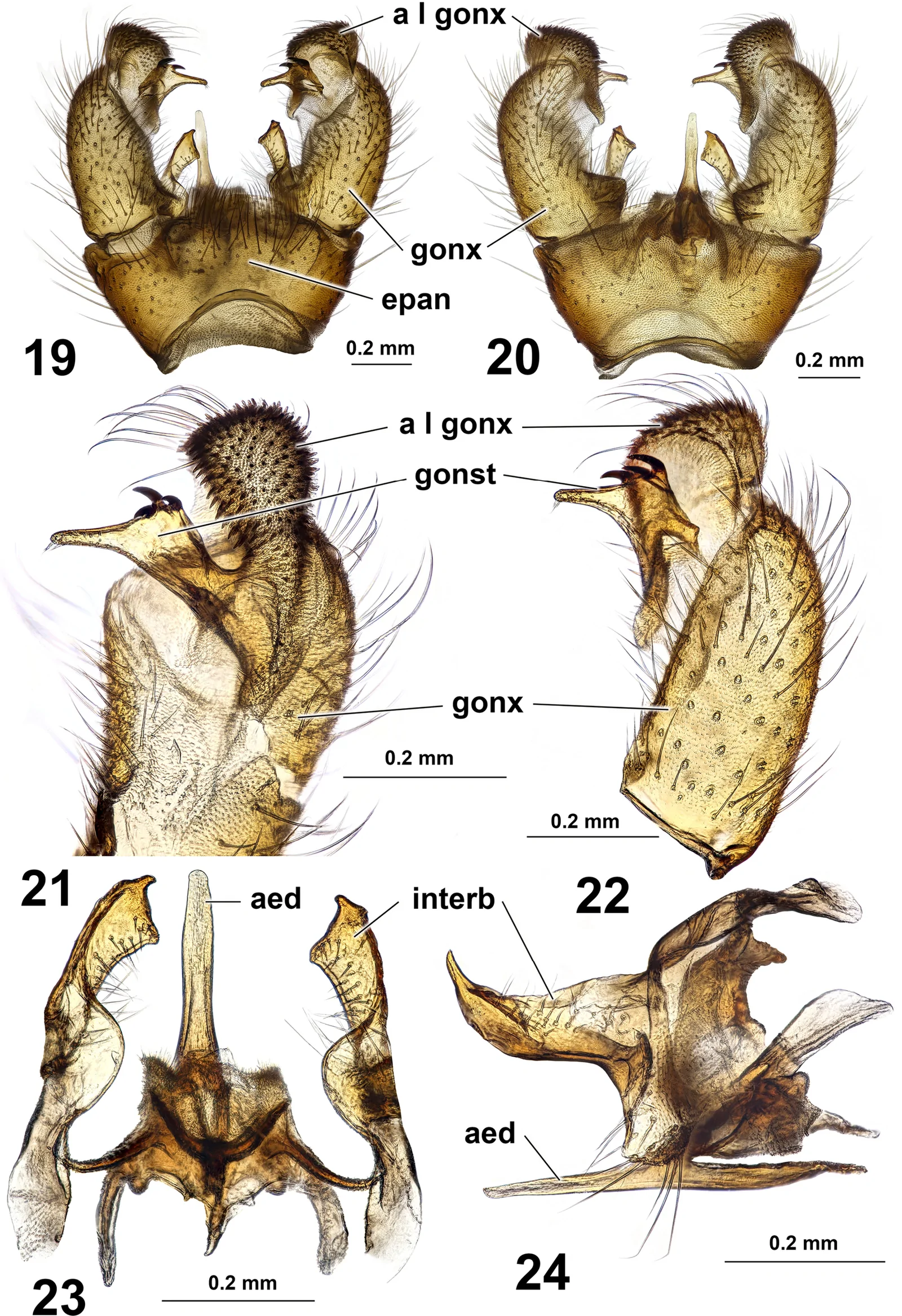
Male terminalia of *Nipponomyia kalbongsanica* Podenas & Podeniene, sp. nov., paratype. **19**. Male genitalia, dorsal view; **20**. Male genitalia, ventral view; **21**. Distal part of gonocoxite and gonostylus, mesal view; **22**. Gonocoxite and gonostylus, dorso-lateral view; **23**. Aedeagal complex, dorsal view; **24**. Aedeagal complex, lateral view. Abbreviations: a l gonx = apical lobe of gonocoxite, aed = aedeagus, epan = epandrium, gonst = gonostylus, gonx = gonocoxite, interb = interbase.

**Figures 25–27.**
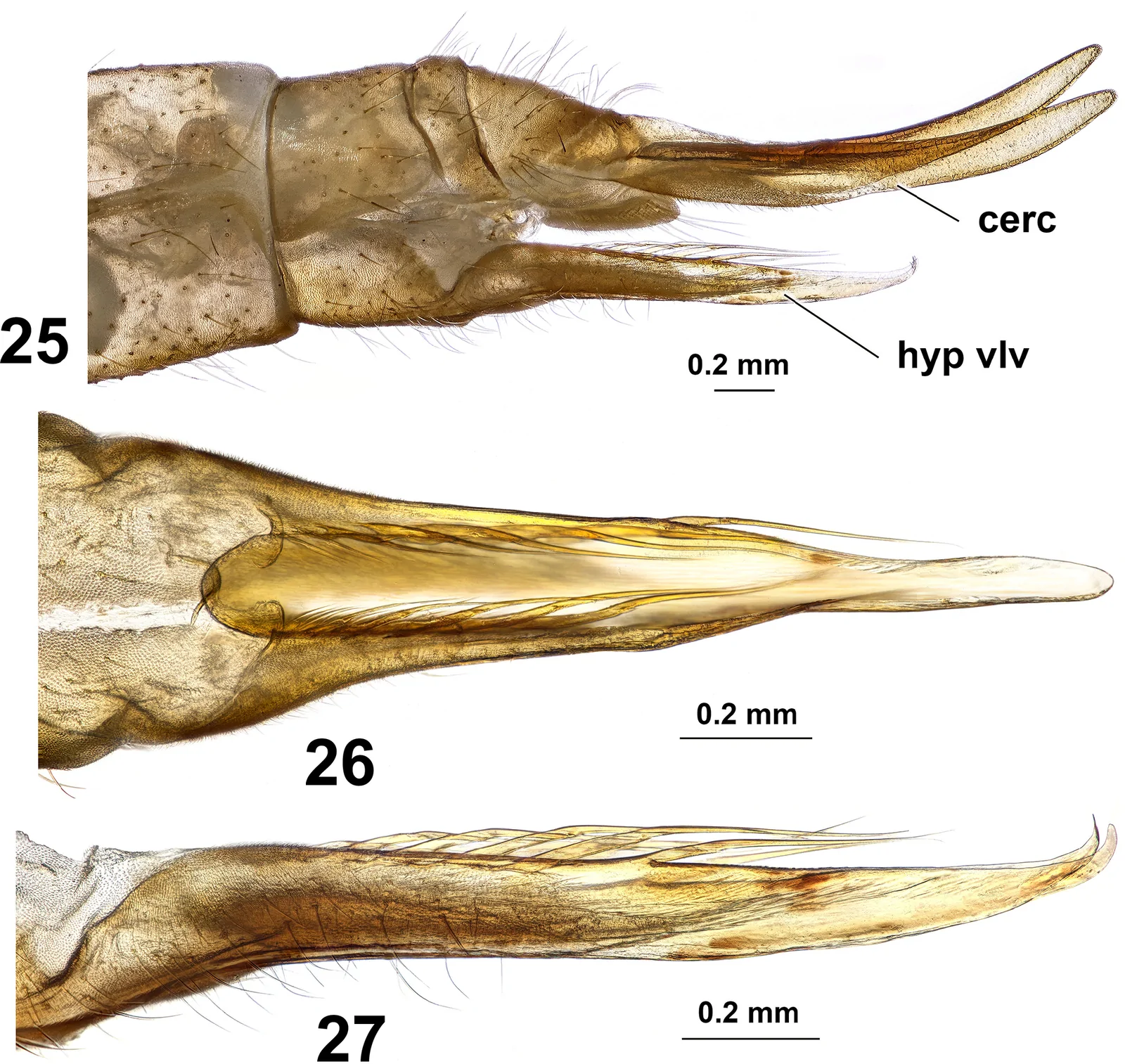
Female terminalia of *Nipponomyia kalbongsanica* Podenas & Podeniene, sp. nov., paratype. **25**. Ovipositor, lateral view; **26**. Hypogynial valvae, dorsal view; **27**. Hypogynial valve, lateral view. Abbreviations: cerc = cercus, hyp vlv = hypogynial valve.

***Abdomen***. Male terminalia (Figs 19, 20) enlarged, slightly wider than preceding abdominal segments. Epandrium distinctly wider than longer, comparatively simple, posterior margin slightly raised and extended caudally, slightly rounded without additional projections; postero-dorsal area densely setose. Posterior margin of hypandrium nearly straight, without projections. Epandrium and hypandrium fused, forming a complete genital ring. Gonocoxite elongate, ~2× as long as wide, usually with a small subbasal enlargement mesally. Apical lobe of the gonocoxite (sometimes referred to as a separate gonostylus) wide and fleshy, covered with abundant small dark spines (Figs 21, 22). Interbase (Fig. 23) large, sometimes with darkened subapical spines. A single pair of gonostyli (the apical lobe of the gonocoxite is sometimes also referred to as a gonostylus). Gonostylus (Figs 21, 22) with a long rostral lobe bearing a few large subbasal teeth. Aedeagus (Figs 23, 24) long and straight, paramere short but bearing a long lateral branch. Ovipositor with a long narrow and arched cercus (Fig. 25), tip raised upwards, apex blunt. Hypogynial valve (Figs 26, 27) straight and narrow, dorsal margin with a few long setae pointing caudally; apex of the valve reaching to about the middle of the cercus. Spermathecae three, small, subglobular.

**Larva**. Overall appearance typical for the subfamily Pediciinae. Body covered with short white pubescence. General appearance of the head capsule as in other Pediciinae: well sclerotised, dorsally depressed, narrow, and elongate in shape with curved dorsal sutures and a slightly curved frontoclypeal suture. Antenna short with two long apical papillae. Mandible large, sickle-shaped with well-developed three dorsal and four ventral teeth. Maxilla sclerotised and consisting of well-developed inner (galea) and outer (palpiger) lobes, and with an elongated, distinctly arched cardo at the base. Hypostoma consisting of two plates, each bearing three teeth. Push-button connection at the base of the innermost teeth not visible. Abdominal segments IV–VII with inconspicuous ventral creeping welts. Terminal segment with two spiracular lobes, which are almost 3× as long as their basal width. Anus surrounded by four white, elongated, fleshy and segmented anal papillae. Spiracular field bare, not covered with setae. Spiracles small, oval in shape, close to each other and at the same level as the spiracular field.

The genus includes only 17 species worldwide (Oosterbroek 2026), 11 of which are Oriental in distribution, while six occur in the East Palaearctic. The genus has no recognised subgenera, is unknown from other biogeographic regions, and is not represented in the fossil record (Evenhuis 2014). Japanese species have been grouped into two morphological species groups, the *N. kuwanai* and *N. trispinosa* species groups (Kolcsár et al. 2020). The *N. kuwanai* species group is characterised by the presence of dark transverse dashes in the costal cell of the wing. The *N. trispinosa* species group lacks transverse dashes in the costal cell and is characterised by a comparatively shorter last palpomere, a gonocoxite with a more prominent ventro-basal lobe, and an acute aedeagus. Non-Japanese species can also be assigned to these morphological species groups, but with some reservations. Thus, the *N. kuwanai* species group includes *N. joshii* (Fig. 2), *N. kamengensis* (Fig. 3), *N. kulingensis* (Fig. 5), *N. kuwanai* (Figs 6, 7), *N. nigrocorporis* (Fig. 9), *N. novempunctata* (Fig. 10), *N. okinawensis*, *N. pentacantha* (Fig. 11), *N. symphyletes* (Fig. 12); however, the small spots in the costal cell of *N. kamengensis*, *N. kulingensis*, and especially *N. nigrocorporis* more closely resemble dots than “typical” transverse dashes. The *N. trispinosa* species group includes *N. flavicollis* (Fig. 1), *N. gracilis*, *N. khasiana* (Fig. 4), *N. mannheimsiana* (Fig. 8), *N. sumatrana*, *N. szechwanensis* (Fig. 13), *N. trispinosa* (Fig. 14), *N. yakushimensis*; however, the costal cells of *N. khasiana* and *N. szechwanensis* have three larger transverse spots, which are absent in *N. trispinosa*.

### Nipponomyia kalbongsanica

Taxon classificationAnimaliaDipteraPediciidae

Podenas & Podeniene sp. nov.

4D170FBF-A435-59F0-A0F6-B95AF4899A3D

https://zoobank.org/069319A5-DEEA-4AAD-B3AC-D94C59565067

Figs 16, 17, 18, 19, 20, 21, 22, 23, 24, 25, 26, 27, 28, 29, 30, 31, 32, 33, 34, 35, 36, 37, 38, 39, 40

#### Type material.

(Fig. 15) **South Korea** • ***Holotype***: (Fig. 16) ♂ (pinned); Gyeonggi-do, Gapyeong-gun, Gapyeong-eup, Gyeongbanan-ro, Kalbongsan Natural Recreational Forest; 37.83667°N, 127.46500°E; alt. 170 m; 21 June 2025 (2); S Podenas leg.; NIBR. ***Paratypes***: • 1 ♀ (pinned); #9, Central National Forest, 29 km NE Seoul [Gyeonggi-do, Pocheon-si, Soheul-eup, Jikdong-ri, Korea National Arboretum]; [37.74946°N, 127.16385°E]; alt. 122–152 m; 29 May 1954; GW Byers leg.; SMEK • 1 ♂ (pinned, genitalia in microvial with glycerol on the same pin); #17, Central National Forest, 29 km NE Seoul; alt. 107–152 m; 20 June 1954; GW Byers leg.; SMEK • 1 ♂ (pinned); #20, Central National Forest Area [Gyeonggi-do, Namyangju-si, Jinjeop-eup]; [37.747189°N, 127.18075°E]; 11 July 1954; GW Byers leg.; USNM • 1 ex. (pinned, abdomen broken); #20, 3 km S. Pup’yong-ni, 26 km NE Seoul [Gyeonggi-do, Namyangju-si, Jinjeop-eup, Bupyeong-ri]; [37.72192°N, 127.18070°E]; alt. 91–137 m; 11 July 1954; GW Byers leg.; SMEK • 1 ♂ (pinned); #24, Central National Forest, 29 km NE Seoul; alt. 122–152 m; 27 July 1954; GW Byers leg.; SMEK • 2 ♂, 1 ♀ (pinned); #25, Central National Forest, 29 km NE Seoul; alt. 122–152 m; 14 August 1954; GW Byers leg.; SMEK • 1 ♂ (pinned); Gyeonggi-do, Gapyeong-gun, Buk-myeon, Hwaak-ri; 37.98402°N, 127.52676°E; alt. 579 m; 20 August 2014 (2); S Podenas, S Kim leg.; NIBR • 1 ♂ (pinned); Gyeonggi-do, Gapyeong-gun, Buk-myeon, Jeokmok-ri; 38.02982°N, 127.44310°E; alt. 560 m; 20 August 2014 (3); S Podenas, S Kim leg.; NIBR • 1 ♂, 1 ♀ (pinned); Jeollanam-do, Gurye-gun, Toji-myeon, Naeseo-ri, Piagol valley; 35.26590°N, 127.58096°E; alt. 446 m; 24 August 2014 (1); S Podenas leg.; net; NIBR • 1 ♂ (pinned, genitalia in microvial with glycerol on the same pin); Jeollanam-do, Gurye-gun, Toji-myeon, Naeseo-ri, Piagol valley; 35.25550°N, 127.58269°E; alt. 284 m; 25 April 2015 (1); S Podenas leg.; NIBR • 1 ♂ (pinned, genitalia in microvial with glycerol on the same pin); Jeollanam-do, Gurye-gun, Toji-myeon, Naeseo-ri, Piagol valley; 35.27177°N, 127.57146°E; alt. 490 m; 27 April 2015 (2); S Podenas leg.; NIBR • 3 ♂ (pinned, genitalia of 1 ♂ in microvial with glycerol on the same pin, wing slide mounted); Jeollanam-do, Gurye-gun, Toji-myeon, Naeseo-ri, Piagol valley; 35.26590°N, 127.58096°E; alt. 446 m; 26 June 2015; S Podenas leg.; NIBR • 1 ♂ (pinned); Jeollanam-do, Gurye-gun, Toji-myeon, Naeseo-ri, Piagol valley; 35.27177°N, 127.57146°E; alt. 490 m; 27 June 2015 (1); S Podenas leg.; NIBR • 1 ♂ (pinned); Jeollanam-do, Gurye-gun, Toji-myeon, Naeseo-ri, Piagol valley; 35.27177°N, 127.57146°E; alt. 490 m; 28 June 2015 (1); S Podenas leg.; NIBR • 1 ♂ (pinned); Jeollanam-do, Gurye-gun, Toji-myeon, Naeseo-ri, Piagol valley; 35.27177°N, 127.57146°E; alt. 490 m; 28 June 2015 (2); S Podenas leg.; NIBR • 3 ♂, 1 ♀ (pinned), 2 ♂, 1 ♀ (in ethanol, wings slide-mounted); Jeollanam-do, Gurye-gun, Toji-myeon, Naeseo-ri, Piagol valley; 35.27177°N, 127.57146°E; alt. 490 m; 29 June 2015 (1); S Podenas leg.; NIBR; GenBank Accession No: PZ235368 • 2 ♂, 1 ♀ (in ethanol); Jeollanam-do, Gurye-gun, Toji-myeon, Naeseo-ri, Piagol valley; 35.25825°N, 127.58208°E; alt. 310 m; 29 June 2015 (2); S Podenas leg.; NIBR • 1 ♂, 1 ♀ (pinned); Jeollanam-do, Gurye-gun, Toji-myeon, Naeseo-ri, Piagol valley; 35.27177°N, 127.57146°E; alt. 490 m; 2 July 2015 (2); S Podenas leg.; NIBR • 2 ♂, 2 ♀ (pinned, ♂ genitalia in microvials with glycerol on the same pins); Gyeongsangbuk-do, Gyeongju, Jinnhyeon-dong; 35.78755°N, 129.34274°E; alt. 320 m; 27 May 2016 (1); S Podenas, H Baek leg.; NIBR • 1 ♂ (in ethanol); Jeollanam-do, Gurye-gun, Toji-myeon, Naeseo-ri, Piagol valley; 35.27177°N, 127.57146°E; alt. 490 m; 3 June 2016 (2); S Podenas leg.; NIBR • 1 larva (in ethanol); Gyeongsangnam-do, Hadong-gun, Hwagae-myeon, Daeseong-ri; 35.29953°N, 127.63350°E; alt. 547 m; 27 June 2019 (2); V Podeniene leg.; NIBR; GenBank accession no. PZ235369 • 4 ♂, 3 ♀ (pinned); Gyeonggi-do, Gapyeong-gun, Gapyeong-eup, Gyeongbanan-ro, Kalbongsan Natural Recreational Forest; 37.83667°N, 127.46500°E; alt. 170 m; 21 June 2025 (2); S Podenas leg.; NIBR.

#### Diagnosis.

Yellow species with contrasting, narrow black fronto-lateral margins of abdominal tergites, with white tibiae and basitarsi. The wing is translucent, with a yellow stripe along the anterior margin, surrounded by small longitudinal dark brown dashes; a brownish area surrounding the cord, and an oblique brownish stripe extending to the distal margin of the discal cell nearly reaching the wing margin. Epandrium of male terminalia with a nearly straight posterior margin, gonocoxite with a large rounded apical lobe covered with abundant small spines, gonostylus with a long narrow rostral prolongation and three large spines on the dorsal (caudal) margin. Interbase large, plate-shaped, curved at the base, aedeagus long, extending beyond the tip of the interbase.

#### Description.

General body colouration is yellow (Fig. 16). Body length of the male 10.1–14.3 mm, of the female 11.6–15.7 mm. Wing length of the male 8.8–11.7 mm, of the female 8.9–11.3 mm.

***Head***. Brownish yellow, sparsely dusted with grey, especially anteriorly, covered with rather dense yellowish setae. Eyes widely separated, the distance between them at the base of the antenna approximately equals the length of the scape. Vertex with a small tubercle, the tip of which in some specimens bears a small dark spot resembling an ocellus. Antenna (Fig. 17) short, 0.9–1.1 mm long in the male, 1.0–1.2 mm in the female, reaching only the frontal margin of the prescutum when bent backwards. Scape elongate, brown with a yellowish distal part, sparsely dusted with grey; pedicel obscure yellow to brownish yellow, elongate, nearly as long as the scape, slightly dilated ventrally, covered with a few yellowish setae. Flagellum 12-segmented, greyish yellow, flagellomeres, except the apical one, short, just slightly longer than wide, covered with whitish pubescence, longest verticils up to 2× as long as the respective segments. Apical segment ~3× as long as the preceding one, probably composed of two fused segments, the 12^th^ and 13^th^, darker than the other flagellomeres, apex with three long verticils approximately as long as the apical flagellomere itself. Rostrum short, dark brown, sparsely dusted with greyish brown. Palpus dark brown except for the pale brown distal palpomere, covered with long yellowish setae. Labellum dark brown.

***Thorax***. Semi-polished brownish yellow. Cervical sclerite brown dorsally, turning pale postero-ventrally. Pronotum yellow with a pale postpronotum, posterior margin with a line of strong, arched, erect brownish setae. Presutural scutum yellow with four indistinct longitudinal semi-polished stripes, not darker than the surrounding matte yellow area, sparsely covered with pruinosity. Posterior margin of the presutural area with six strong erect dark brown setae. Tubercular pit missing, pseudosutural fovea slightly paler than the surrounding area. Prothoracic spiracle surrounded by a whitish membrane. Scutal lobe yellow, area between the scutal lobes concolourous with the scutal lobe. Scutellum pale yellow. Mediotergite brownish yellow. Pleuron brownish yellow, paler ventrally. Metathoracic spiracle surrounded by a white rim. Wing (Fig. 18) comparatively broad for the genus, 3.3× as long as wide, similar in both sexes. Wing pattern with a distinct longitudinal yellow stripe along vein *R*, bordered by brownish shading in cells *c* and *r*, and interrupted by diffuse dark brown marks. A dark brown spot with a yellowish central area extends from *R_2_* through *R_3_*, the distal part of *R_4_*, and crosses the tip of *R_5_*. A narrower dark stripe extends from the base of *R_2+3_* through *m-m* and reaches the wing margin in some specimens but extends just a short distance beyond *M_3_* not reaching the wing margin in others. Dark areas surround *r-m* and *m-cu*. Most veins are brownish, veins *Sc*, *R*, *Cu* and *A_1_* are yellow. Venation: bases of veins *R*, *M* and *Cu* reach each other, thus the *arculus* is very short or missing, vein *Sc* long, reaching the wing margin at the branching point of *R_2+3_* and *R_4_*, *sc-r* located a long distance before the base of *Rs*; *R_1_* 2.2× as long as *R_2_*. Radial sector long, 4.5× as long as *m-cu*, branching into *R_2+3+4+5_* and *r-m*. Cell *r_4_* with a short stem. Discal cell very long, nearly 5× as long as wide. Cell *m_1_* without a stem, cross vein *m-m* on *M_2_. M_4_* arising before the middle of the discal cell. Cross-vein *m-cu* at the branching point of *M.* Veins *CuP* and *A_1_* strongly divergent, *CuP* nearly straight, *A_1_* slightly arched at the apex and reaching the wing margin approximately at the level of the base of *Rs.* Anal cell long and narrow. Wing squama with a few setae. Halter long, whitish with a slightly infuscate distal margin of the knob. Length of the male halter 1.3–1.5 mm, of the female 1.7–1.8 mm. Coxae yellow, fore coxa somewhat paler. Trochanters yellow to pale yellow. Male femora yellow with indistinctly infuscate apices, female femora pale. Tibiae whitish with narrow dark brown tips. Basitarsi whitish basally, turning yellow distally, remaining tarsomeres turning dark brown towards the apex. Male femur I: 5.7–6.1 mm long, II: 5.8–7.3 mm, III: 6.4–7.6 mm, tibia I: 6.1–6.7 mm, II: 5.8–7.2 mm, III: 6.4–7.9 mm, tarsus I: 8.1–9.0 mm, II: 6.1–7.6 mm, III: 7.1–8.3 mm. Female femur I: 5.8 mm long, II: 6.0 mm, III: 5.6–6.6 mm, tibia I: 5.8 mm, II: 6.0 mm, III: 6.0–6.5 mm, tarsus I: 6.1 mm, II: 6.2 mm, III: 5.5–5.7 mm. Claw just slightly arched, simple, without spines, arolium distinct.

***Male abdomen***. Tergites brownish yellow, covered with erect dark brown setae; sternites pale yellow, covered with erect pale-yellow setae. Tergites II–VI with a distinct narrow dark brown to black line along the anterolateral margin; sternites II–V each with a dark spot below that line. The spots on sternites II and III are elongate, whereas those on sternites IV and V are triangular. Pregenital segment and male terminalia (Figs 16, 19, 20) brownish. Epandrium simple, distinctly wider than long; posterior margin evenly convex, slightly raised and extended caudally, without additional projections, and covered posterocaudally with long, dense setae. Posterior margin of hypandrium nearly straight, without projections. Epandrium and hypandrium fused, forming a complete genital ring. Gonocoxite elongate, 2.2× as long as wide, with a rather prominent ventrobasal lobe. Apical lobe of gonocoxite (Figs 19–22, a l gonx) large and rounded, with a long posteromesal lobe; entire lobe covered with numerous small dark spines, especially dense caudally. Apical lobe with a narrow membranous area separating it from the rest of the gonocoxite (Fig. 20); therefore, it more closely resembles the outer lobe or branch of the gonostylus than an extension of the gonocoxite (Fig. 21). Gonostylus itself (or inner branch of the gonostylus, depending on interpretation) with a long rostral lobe bearing a few fine setae apically (Figs 21, 22); caudal margin with a low hump bearing three large, black, slightly arched spines. Interbase large, plate-shaped, with the caudal angle slightly extended, the basal portion widened and convex, and the mesal surface covered with fine setae (Fig. 23). Aedeagus (Figs 23, 24) long, narrow, straight, and rod-shaped, reaching approximately the tip of the interbase. Paramere short but bearing a long posterolateral branch. Seminal vesicle small.

***Female abdomen***. Pale yellow, with a dark pattern similar to that of the male; posterior segments and ovipositor (Fig. 25) brownish, tip of the cercus pale. Cercus long and narrow; distal part only slightly upcurved, apex blunt. Hypogynial valve (Figs 26, 27) long, narrow, and straight, with only the very tip distinctly upcurved. The tip of the valve reaches slightly beyond the middle of the cercus; dorsal margin with five short, fine setae basally and five long, strong setae with enlarged bases, directed caudally but not reaching the tip of the valve. Bases of the strong setae are situated on the basal half of the dorsal margin. Distal part of sclerite bare, without setae. Spermathecae three, small, subglobular.

**Figures 28–32.**
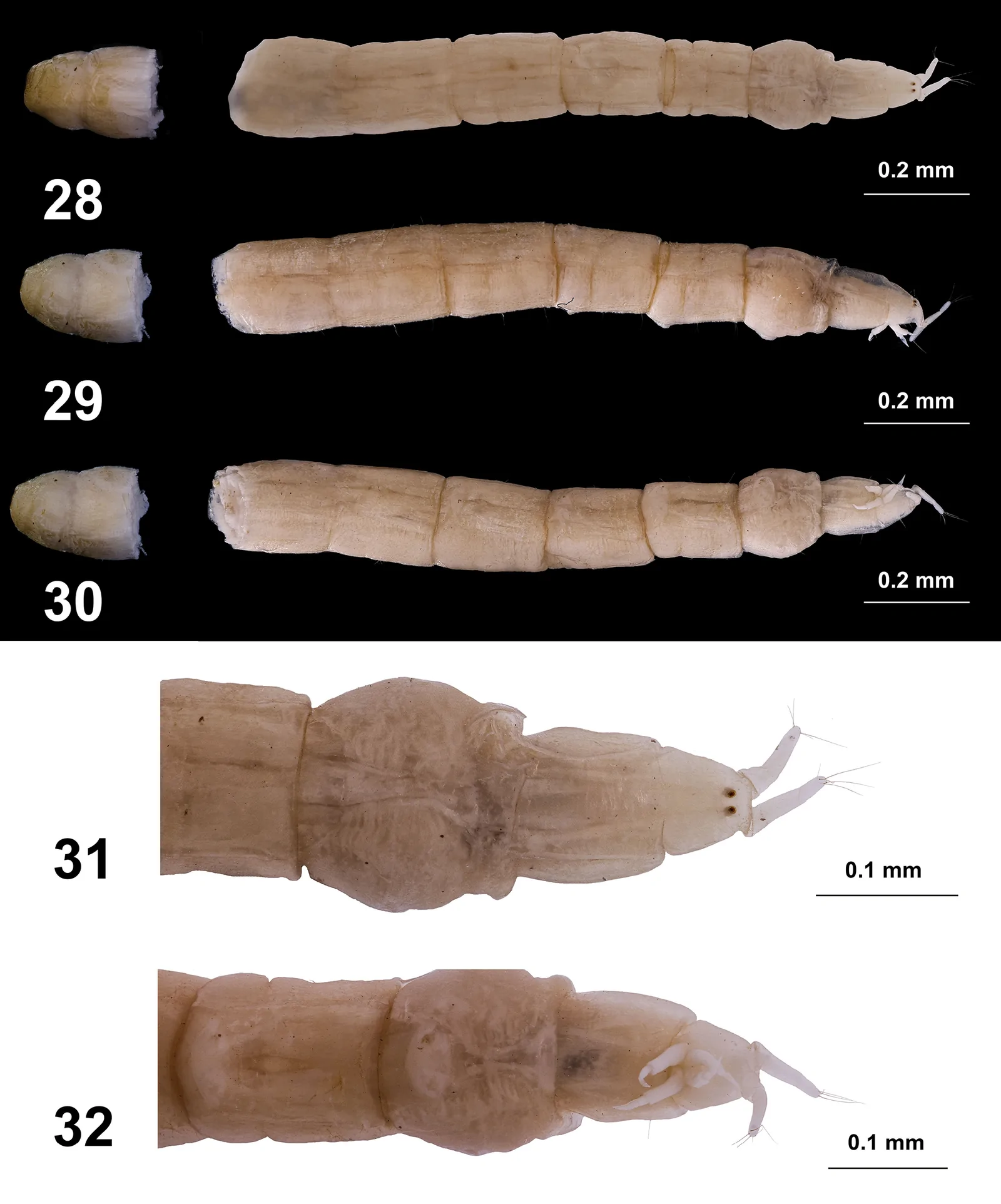
*Nipponomyia kalbongsanica* Podenas & Podeniene, sp. nov. **28**. Larva, dorsal view; **29**. Larva, lateral view; **30**. Larva, ventral view; **31**. Spiracular field; **32**. Anal field.

**Larva**. Last-instar larvae. Length 15.8 mm; width 2.6 mm. Larvae hemicephalic, metapneustic (Figs 28–32). Body covered with short white pubescence, which gives the body a whitish colour. The first thoracic segment and abdominal segments III–VII are longer than wider. The length of the two remaining thoracic segments and the first two abdominal segments exceeds their width. Abdominal segments IV–VII with inconspicuous ventral creeping welts. Creeping welts on segments V–VII considerably wider than on segment IV, and their width increases gradually towards the posterior end (Figs 29, 30). Their apical part bears short, pale spinulae.

**Figures 33–40.**
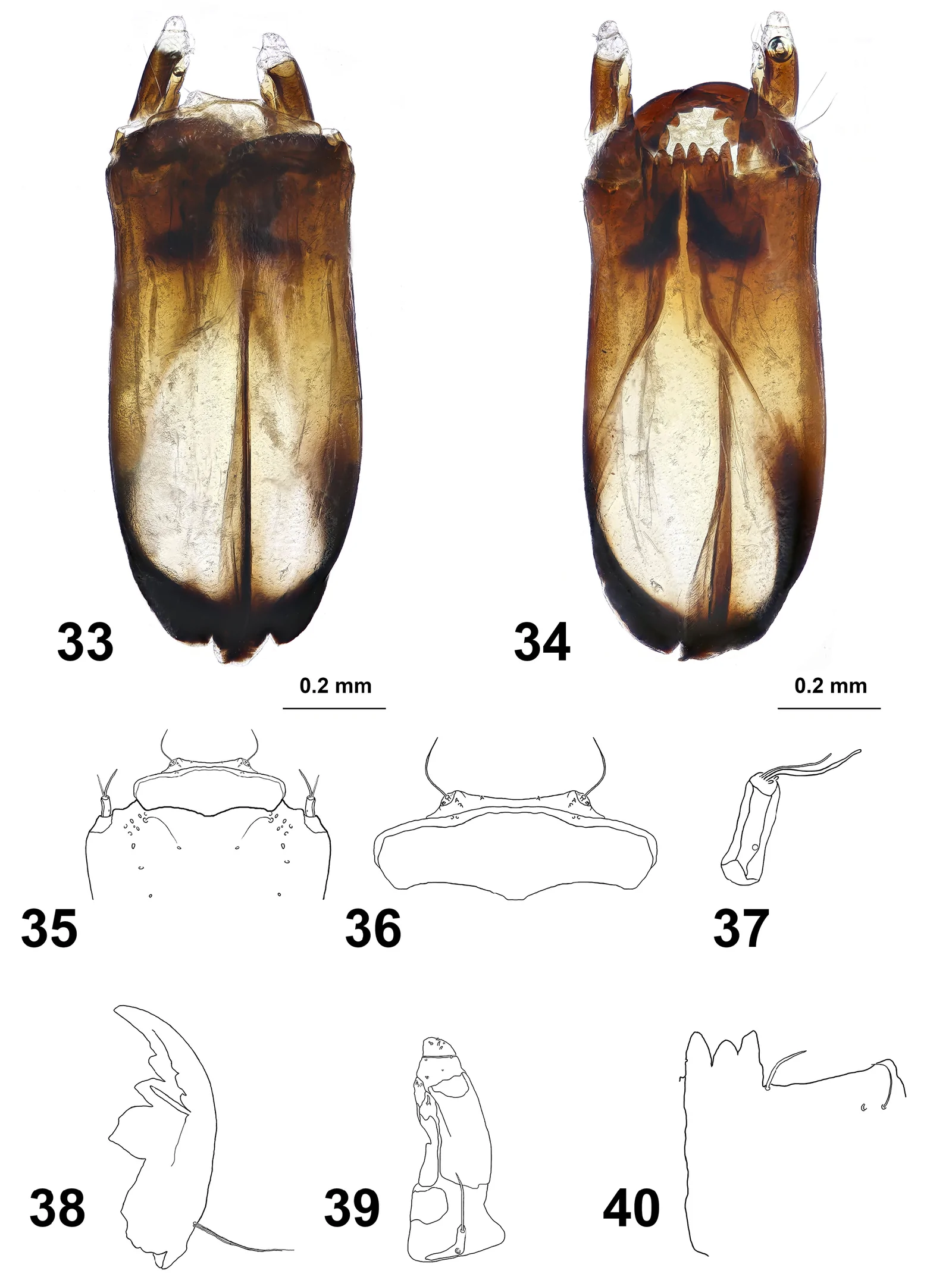
*Nipponomyia kalbongsanica* Podenas & Podeniene, sp. nov. **33**. Head capsule, dorsal view; **34**. Head capsule, ventral view; **35**. Labrum, clypeus, and uppermost part of the frons; **36**. Labrum and clypeus; **37**. Left antenna, dorsal view; **38**. Right mandible, dorsal view; **39**. Right maxilla, ventral view; **40**. Part of hypostoma, left side.

***Head capsule***. Length 1.3 mm, width 0.47 mm. Well-sclerotised, massive, dorsally depressed, narrow, and elongate in shape, partially retracted within the thorax. Lateral margins of the head capsule parallel, posterior parts of the genae are heavily sclerotised (Figs 33, 34). Dorsal sutures short, arched and directed outward. Frontoclypeal suture straight with lateral parts raised. There are two very long setae at the base of each side. Six sensory pits are located around them and one pit near the middle line (Fig. 35). Two sensory pits are located on either side near the middle of the frons (Figs 35, 36). Clypeus wide and trapezoidal, with rounded margins. Labrum saddle-shaped with several sensory structures: one long seta, five short sensory papillae and two pits on the apical part on each side, a short papilla present in the middle (Fig. 36). Epipharynx covered with short dense bristles. Antenna short, reaching to about one fifth of the length of the mandible (Fig. 33). Basal segment cylindrical, twice as long as wide, with sensory pit near the base. There are two very long (slightly longer than the basal segment itself) apical papillae (the inner seta is one third longer than the outer seta) and two very short sensory structures on the apex of the basal segment of the antenna (Fig. 37). Mandible large, heavily sclerotised and sickle-shaped, with three dorsal and four ventral teeth, the apical tooth being the largest (Fig. 38). The first dorsal tooth is very small. The second dorsal tooth is triangular in shape and well developed. The third dorsal tooth is triangular and more than twice as long as the second tooth. The fourth dorsal tooth is large, more than twice as long as the third tooth, triangular in shape, with the apex abruptly narrowed and sharply pointed. Three ventral teeth small and blunt, the fourth tooth long and acute. There are two long setae at the base of the mandible. Maxilla well developed and consist of an outer lobe (palpiger) and inner lobe (galea) (Fig. 39). Palpiger is cylindrical and more prominent, with a large sclerite at the base. Its apical part surrounded by a sclerotised ring. Palpus button-shaped, with a few sensory structures (exact number difficult to determine). There are two sensory pits on the outer margin and one sensory pit on the inner margin of the apical part of the palpiger. Galea finger-shaped, with a large sclerite at the base. Its apical part sclerotised, bearing two long setae and one papilla. Cardo arched, well developed with very long setae and a sensory pit. Hypostoma heavily sclerotised, divided into two plates each of which bears three sharp teeth (Figs 34, 40). Connection between the two plates of the hypostoma without clear separation into push-buttons. The innermost and outermost teeth are similar in size; the middle tooth is significantly shorter. Prementum a flattened plate with protruding lateral lobes and apically bearing short teeth.

***Spiracular disc***. Spiracular disc bears two conical, elongate ventral lobes, each almost 3× as long as its basal width, apically pointed with four long and one medium-long seta on the apex (Fig. 31). Spiracles very small, oval in shape. The distance between them is almost equal to the width of a spiracle. Spiracular disc not covered with setae.

***Anal field***. Anus surrounded by four long, segmented, white and fleshy anal papillae (Fig. 32). Papillae finger-shaped and equal in size, almost the same length as the spiracular lobes.

#### Elevation.

From ~ 100–600 m.

#### Period of activity.

Adults fly from late April through the end of August.

#### Habitats.

The larva was collected from mud accumulated on stones beneath leaf litter in a stream. Adults fly close to small fast running springs, small and medium-sized mountainous streams running through rocks, covered with mosses or algae. Usually these streams are surrounded by mixed forests or just pine groves, with sparse grassy vegetation on the ground. They were also found flying around a small spring on a mountainous slope under pine and oak groves.

#### Distribution.

Currently known only from South Korea (Table 1).

**Table 1.** Collecting sites in South Korea.

**Locality**	**Year**	**Latitude, Longitude**	**Collector**	**Method**	**Depository**
Central National Forest, 29 km NE Seoul [Gyeonggi-do, Pocheon-si, Soheul-eup, Jikdong-ri, Korea National Arboretum]	1954	37.74946°N, 127.16385°E	GW Byers	Net	SMEK
Central National Forest Area [Gyeonggi-do, Namyangju-si, Jinjeop-eup]	1954	37.74719°N, 127.18075°E	GW Byers	Net	SMEK
3 km S. Pup’yong-ni, 26 km NE Seoul [Gyeonggi-do, Namyangju-si, Jinjeop-eup, Bupyeong-ri]	1954	37.72192°N, 127.18070°E	GW Byers	Net	SMEK
Gyeonggi-do, Gapyeong-gun, Buk-myeon, Hwaak-ri	2014	37.98402°N, 127.52676°E	S Podenas, S Kim	Net	NIBR
Gyeonggi-do, Gapyeong-gun, Buk-myeon, Jeokmok-ri	2014	38.02982°N, 127.44310°E	S Podenas, S Kim	Net	NIBR
Jeollanam-do, Gurye-gun, Toji-myeon, Naeseo-ri, Piagol valley	2014	35.26590°N, 127.58096°E	S Podenas	Net	NIBR
	2015	35.25550°N, 127.58269°E			
	2016	35.27177°N, 127.57146°E			
		35.25825°N, 127.58208°E			
Gyeongsangbuk-do, Gyeongju, Jinnhyeon-dong	2016	35.78755°N, 129.34274°E	S Podenas, H Baek	Net	NIBR
Gyeongsangnam-do, Hadong-gun, Hwagae-myeon, Daeseong-ri	2019	35.29953°N, 127.63350°E	V Podeniene	Sieve	NIBR
Gyeonggi-do, Gapyeong-gun, Gapyeong-eup, Gyeongbanan-ro, Kalbongsan Natural Recreational Forest	2025	37.83667°N, 127.46500°E	S Podenas	Net	NIBR

#### Etymology.

The species is named after the type locality, Kalbongsan Natural Recreational Forest.

##### Remarks

###### Adult

Only six species of *Nipponomyia* are recorded from the Eastern Palaearctic. They belong to two species groups, the *N. kuwanai* group and the *N. trispinosa* group. Based on adult morphology, the new species, together with four other species, belongs to the *N. trispinosa* species group. Two of these species, *N. trispinosa* (Alexander, 1920) and *N. yakushimensis* (Kolcsár & Kato, 2020), can be easily distinguished by the presence of dark spots on the thoracic dorsum. They also differ from *N. kalbongsanica* Podenas & Podeniene, sp. nov. in wing pattern and venation, as well as in male genitalia. *N. gracilis* Savchenko, 1983 and *N. szechwanensis* Alexander, 1935 lack dark spots on the thoracic dorsum, which is brownish yellow to yellow, similar to that of *N. kalbongsanica* Podenas & Podeniene, sp. nov. *N. szechwanensis* is known only from Sichuan Province, China. In *N. szechwanensis*, the wing is narrower and its venation and pattern are distinctly different from those of *N. kalbongsanica* Podenas & Podeniene, sp. nov.; *N. szechwanensis* has a shorter *Rs*, a shorter discal cell, cross-vein *m-m* transverse and reaching *M_2_* slightly beyond its base, and the dark stripe extending along the distal margin of the discal cell reduced to a dark spot at the base of *R_2+3_*. The male genitalia of *N. szechwanensis* have a lower apical lobe of the gonocoxite and a very different interbase, which tapers towards the apex, whereas in *N. kalbongsanica* Podenas & Podeniene, sp. nov. the interbase is wide and flat. *N. gracilis* is known from the Russian Far East, close to the border with North Korea, and was described from a single specimen. In general, it resembles *N. kalbongsanica* Podenas & Podeniene, sp. nov., but differs in the wing, male terminalia, and other structures. It has a 15-segmented antenna, whereas that of *N. kalbongsanica* Podenas & Podeniene, sp. nov. is 14-segmented, although the distal segment is probably formed by the fusion of two apical flagellomeres. The wing of *N. szechwanensis* is narrower; it has a shorter *Rs* and dark spots at the tips of *M*, *CuA*, and *CuP* on the wing margin, which are absent in *N. kalbongsanica* Podenas & Podeniene, sp. nov. The male genitalia of *N. szechwanensis* have the caudal margin of the gonostylus slightly swollen, without a hump, and bear only two large black teeth, whereas those of *N. kalbongsanica* Podenas & Podeniene, sp. nov. have a hump armed with three strong spines. The distal part of the interbase of *N. szechwanensis* resembles a bird’s head, with two strong black teeth, whereas in *N. kalbongsanica* Podenas & Podeniene, sp. nov. it is wide, flat, and plate-shaped. The antennal scape is darker in *N. kalbongsanica* Podenas & Podeniene, sp. nov., and the tibiae and basitarsi are whitish, whereas in *N. szechwanensis* they are yellow. In the key of Kolcsár et al. (2020), the new species runs to couplet 7. It differs from *N. szechwanensis* by having darkened tips of the tibiae. A gonostylus with three spines and the femora are uniformly yellow in both species.

###### Larva

The overall appearance of the larva is similar to that of *Heterangaeus* and *Tricyphona*. The body setae vary in length and colouration within Pediciinae, but in *Nipponomyia* the entire body is covered with very short white pubescence. This character distinguishes it from *Heterangaeus*.

####### Head

The general structure of the larval head capsule of *Nipponomyia* is similar to that of other genera in the subfamily, with differences evident in several details. In most known larvae of Pediciinae, the dorsal sutures are short and straight, whereas in *Nipponomyia* and *Heterangaeus* they are curved and directed outward. The frontoclypeal suture of *Nipponomyia* is slightly curved, more curved than in *Pedicia* and *Tricyphona*, but less so than in *Dicranota*. The labrum is saddle-shaped in all Pediciinae, but the number and arrangement of sensory structures differ among genera and species. Two types of antennal length occur in the subfamily: long antennae reaching the apical part of the labrum, and short antennae extending only slightly beyond the base of the mandible. The antennae of *Nipponomyia*, as well as those of *Heterangaeus*, *Nasiternella*, and *Pedicia*, belong to the latter type. Within Pediciinae, the number and length of apical antennal papillae vary among species within the same genus and therefore cannot be used for generic diagnosis. Mandibles of Pediciinae larvae are large and generally similar in shape. Four ventral teeth of varying sizes and shapes are present in all known species. The dorsal teeth may be well developed or inconspicuous. *Nipponomyia*, as well as *Heterangaeus*, *Tricyphona*, and *Nasiternella varinervis*, possesses three well-developed dorsal teeth. The structure of the maxilla is generally similar throughout Pediciinae. Differences occur in the size of the cardo and in the degree of reduction of the galea. The cardo is less reduced in *Nipponomyia*, *Heterangaeus*, and *Pedicia* than in *Dicranota*, *Tricyphona*, and *Nasiternella*. The galea is less reduced in *Nipponomyia*, *Heterangaeus*, *Pedicia*, and *Tricyphona* than in *Dicranota* and *Nasiternella*. The hypostoma of Pediciinae larvae consists of two plates, each bearing three (or occasionally four) teeth. These plates may be connected either by a push-button–like articulation or by a continuous connection without a distinct push-button separation. *Nipponomyia*, *Heterangaeus*, and some species of *Dicranota* and *Pedicia* possess the latter condition.

####### Abdomen

*Nipponomyia*, as well as *Heterangaeus* and *Tricyphona*, possesses four creeping welts on abdominal segments IV–VII. However, in *Nipponomyia* the welts are inconspicuous and only slightly elevated, whereas in *Heterangaeus* and *Tricyphona* all four welts are laterally elevated into distinct humps. Two types of spiracular field occur within Pediciinae. In one type, the spiracular field is entirely covered with long setae; in the other, the area surrounding the spiracles bears short setae, usually much shorter than those covering the spiracular lobes. *Nipponomyia*, as well as *Heterangaeus* and *Pedicia*, belongs to the latter type, with the spiracular field covered with very short setae. In contrast, *Tricyphona*, *Nasiternella regia*, and *Dicranota* possess spiracular fields covered with long setae. In Pediciinae, the spiracles may be situated either on small elevations or at the same level as the spiracular field. In *Nipponomyia*, as well as in *Heterangaeus*, *Tricyphona*, and *Nasiternella*, the spiracles are located at the same level as the spiracular field. All known larvae of Pediciinae have two ventral spiracular lobes, the length of which varies among genera. Spiracular lobes of *Nipponomyia* are similar in length to those of *Heterangaeus*, *Pedicia*, and *Dicranota*, being almost 3× as long as their basal width. In contrast, the spiracular lobes of *Tricyphona* and *Nasiternella* are considerably shorter. Larvae of *Nipponomyia*, *Heterangaeus*, and *Tricyphona* possess four segmented anal papillae, but in *Nipponomyia* these structures are considerably longer.

##### Habitats

Larvae of *Nipponomyia*, *Heterangaeus*, and *Tricyphona* develop in very similar habitats, typically in mud accumulated on stones beneath leaf litter in streams. In contrast, *Dicranota* usually develops in running water in streams and springs, *Pedicia* develops in the benthic zone of springs and *Nasiternella* can be found in rotten wood or water-filled tree holes.

##### Phylogenetic analysis

**Figure 41.**
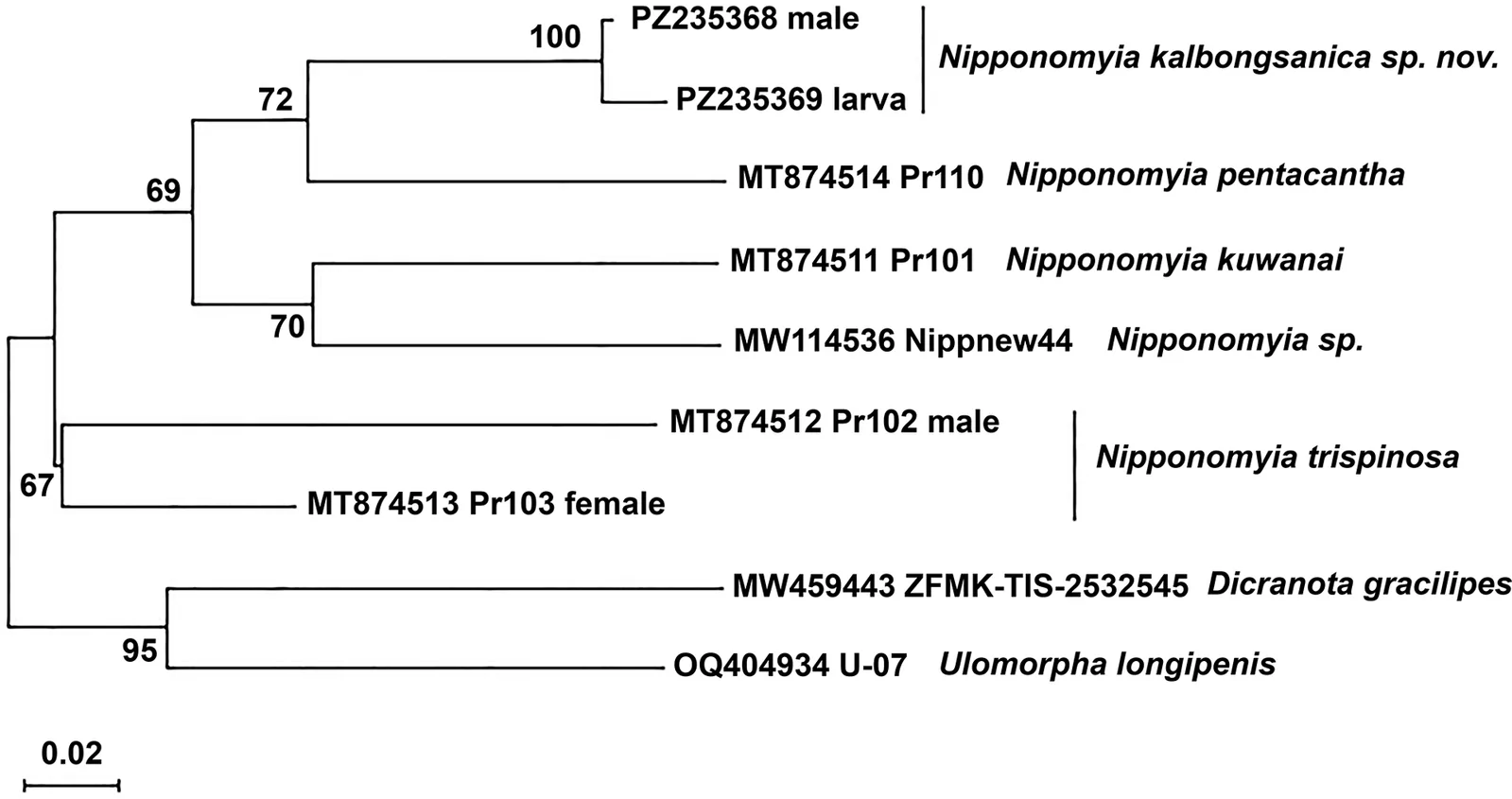
Maximum likelihood phylogenetic tree constructed using mitochondrial COI gene sequences (682 bp) of *Nipponomyia* species with *Dicranota gracilipes* and *Ulomorpha longipenis* as the outgroup. Bootstrap values (1000 replicates) are indicated in the tree. Species names, life stages, and GenBank accession numbers are given.

The South Korean larval specimen and the putative adult specimen were grouped into one cluster in the maximum likelihood (ML) phylogenetic tree (Fig. 41). This association is further supported by the low Kimura 2-parameter (K2P) genetic distance between the sequences (0.0163; Table 2).

**Table 2.** Kimura 2-parameter (K2P) distances.

**Species**	**KR01**	**KR26**	**MT874511**	**MT874512**	**MT874513**	**MT874514**	**MW114536**
KR01							
KR26	0.0163						
MT874511	0.1529	0.17					
MT874512	0.1476	0.1524	0.1450				
MT874513	0.1215	0.1297	0.1430	0.1295			
MT874514	0.1196	0.1296	0.1303	0.1792	0.1266		
MW114536	0.1720	0.1653	0.1542	0.1790	0.1488	0.1656	

Abbreviations: KR01 *Nipponomyia kalbongsanica* Podenas, Podeniene, sp. nov. male, GenBank accession no. PZ235368; KR26 *Nipponomyia kalbongsanica* Podenas, Podeniene, sp. nov. larva, GenBank accession no. PZ235369; MT874511*Nipponomyia kuwanai* voucher Pr101 (Japan: Hokkaido • 1♂; Higashikawa, Asahidake, River Yukomabetsu; alt. 1120 m; 43°39.14'N, 142°48.14'E; 23 Jul. 2019); MT874512*Nipponomyia trispinosa* voucher Pr102 (Japan: [Hokkaido] • 1♂; Hokkaido, Sobetsu, River Benkei; alt. 238 m; 42°33.52'N, 140°59.29'E; 29 Jul. 2019); MT874513*Nipponomyia trispinosa* voucher Pr103 (Japan: [Shikoku] • 1♂, 1♀; Ehime, Toon, Shiraino waterfall; alt. 685 m; 33°45.54'N, 132°58.16'E; 16 Sep. 2019); MT874514*Nipponomyia pentacantha* voucher Pr110 (Japan: [Honshu] • 1♀; Aomori, Nishimeya, Shirakami Nature observation garden, Kawaratai; alt. 255 m; 40°31.13'N, 140°12.89'E; 4 Jul. 2013); MW114536*Nipponomyia* sp. Nippnew44.

Based on morphological evidence, *N. kalbongsanica* Podenas & Podeniene, sp. nov. belongs to the *N. trispinosa* species group, whereas the clustering pattern in the ML tree shows a closer relationship with species of the *N. kuwanai* species group (Fig. 41).

Moreover, both the K2P distances (Table 2) and the ML phylogenetic reconstruction (Fig. 41) indicate that the genetic divergence between the two analysed *N. trispinosa* sequences representing a male and a female is comparable to the interspecific distances observed among other *Nipponomyia* species. This result raises additional questions regarding the taxonomic status and possible hidden diversity within *N. trispinosa*.

## Supplementary Material

XML Treatment for Nipponomyia

XML Treatment for Nipponomyia kalbongsanica

## Additional information

### Artificial Intelligence (AI) use

The authors accept full responsibility for the content of the manuscript, including the disclosure of any use of AI.

Regarding the use of AI in the preparation of this manuscript, the authors declare the following: GPT.

**Used for**: Language, style and writing.

AI was used to improve language.

### Author ORCIDs

S. Podenas https://orcid.org/0000-0002-2597-566X

V. Podeniene https://orcid.org/0000-0002-3807-2347

J. Havelka https://orcid.org/0009-0004-0697-4567

### Data availability

All of the data that support the findings of this study are available in the main text.
